# Participating in a Community of Learners enhances resident perceptions of learning in an e-mentoring program: proof of concept

**DOI:** 10.1186/1472-6920-11-3

**Published:** 2011-01-25

**Authors:** Timona Obura, William E Brant, Fiona Miller, I John Parboosingh

**Affiliations:** 1 Postgraduate Medical Education Director, Aga Khan University, Nairobi, Kenya; 2 Professor of Radiology, Department of Radiology, University of Virginia Health System, Virginia USA; 3 Director of Research, Radiological Society of North America, 820 Jorie Blvd, Oak Brook, IL, 60523, USA; 4 Professor emeritus, University of Calgary, Consultant Community Learning, Calgary, Canada

## Abstract

**Background:**

Community learning and e-mentoring, learning methods used in higher education, are not used to any extent in residency education. Yet both have the potential to enhance resident learning and, in the case of community learning, introduce residents to basic lifelong learning skills. We set out to determine whether residents participating in an Internet based e-mentoring program would, with appropriate facilitation, form a community of learners (CoL) and hold regular community meetings. We also determined resident and faculty perceptions of CoL and Internet sessions as effective learning experiences.

**Methods:**

A six-month e-mentoring pilot was offered to 10 Radiology residents in the Aga Khan University Postgraduate Medical Education Program in Nairobi, Kenya (AKUHN) with a Professor of Radiology, located at University of Virginia, USA, acting as the e-mentor. Monthly Internet case-based teaching sessions were facilitated by the e-mentor. In addition, residents were coached by a community facilitator to form CoL and collectively work through clinical cases at weekly face-to-face CoL sessions.

Event logs described observed resident activity at CoL sessions; exit survey and interviews were used to elicit perceptions of CoL and Internet sessions as effective learning experiences.

**Results:**

Resident adoption of CoL behaviors was observed, including self-regulation, peer mentoring and collaborative problem solving. Analysis revealed high resident enthusiasm and value for CoL. Surveys and interviews indicated high levels of acceptance of Internet learning experiences, although there was room for improvement in audio-visual transmission technologies. Faculty indicated there was a need for a larger multi-specialty study.

**Conclusions:**

The pilot demonstrated resident acceptance of community building and collaborative learning as valued learning experiences, addressing one barrier to its formal adoption in residency education curricula. It also highlighted the potential of e-mentoring as a means of expanding faculty and teaching materials in residency programs in developing countries.

## Background

In e-mentoring, a mentor acts as learner facilitator using virtual technologies as the major methods of communication with mentees. While the benefits of e-mentoring in higher education are reported in the literature, its application in residency education has not been explored [1]. Yet e-mentoring has the potential to expose residents to teachers from other programs and clinical materials not available locally.

### Context

A six-month pilot project was established with author W.E.B., Professor of Radiology, University of Virginia, USA, acting as e-mentor to Radiology residents in the Aga Khan University Postgraduate Medical Education Program in Nairobi, Kenya (AKUHN). The residents met with W.E.B. during a 5-day visit to AKUHN in September 2007 when the educational goal of the pilot, namely, to enhance image interpretation and problem solving skills of Radiology residents was established.

The pilot was designed to meet three education requirements described as *presences *in Garrison's conceptual framework for online courses - *teaching, social *and *cognitive presence *[2]. *Teaching presence*, which includes instructional design, facilitation of learner interactivity, and direct instruction, was undertaken by the e-mentor who was located in Virginia, USA. The e-mentor provided 6 two-hour case-based online teaching sessions between October 2007 and April 2008 to residents located in Nairobi, Kenya.

In addition, residents were invited to form a community of learners (CoL) and meet face-to-face for one hour each week to discuss information acquired during the Internet sessions and collectively explore its relevance to Radiology practice in Kenya. CoL sessions fulfilled the *social presence *in Garrison's conceptual framework which refers to the environment provided for learners to feel safe enough to project themselves socially and emotionally as 'real' people in practice. Garrison's *cognitive presence*, which refers to the extent to which learners are able to construct meaning and make decisions, was fulfilled by a systematic approach to learning from clinical cases taught and role modeled in teaching sessions.

We defined CoL based on Wenger's definition of a community of practice as a group of people who share an interest for what they do and engage in collaborative learning that creates bonds between them [3]. Learning in most group education sessions in residency programs at AKUHN and around the world tend to be teacher-centered and focus on evidence-based explicit knowledge. Discussions in CoL in addition to explicit knowledge include topics such as workarounds and improvisations and clinical judgment typically passed on by storytelling during informal conversations between trusted colleagues. Dornan [4] and Mennin [5] suggest that teaching/learning formats be formally expanded to include communities of learners in which learning to practice is viewed as a social phenomenon and emerges through interactivity and self-organization.

We anticipated that resident participation in CoL would encourage collaborative learning behaviors, including promotive interaction, social skills, group processing and interdependence as well as individual accountability [6]. Studies show that collaborative learning, compared to individual and competitive learning scenarios, brings students to a higher achievement level and raises their problem-solving abilities [6]. Higher education has consistently viewed community as essential to support collaborative learning and discourse associated with higher levels of learning [6]. In addition, participation in community encourages motivation to learn, self-regulation and peer mentoring; lifelong learning behaviors that should be acquired in residency but are not explicitly included in most curricula [7,8].

It was anticipated that residents, graduates of traditional teacher-directed education systems where self is the significant social unit, might not perceive value in learning through participating in a CoL.

### Aims of the pilot

The aims of the pilot were two-fold: we set out to determine whether residents would with assistance from a designated community facilitator form a CoL and demonstrate collaborative learning behaviors; and, obtain resident and faculty perceptions of CoL and Internet sessions as effective learning experiences.

## Methods

The pilot was approved by Ethics Boards at AKUHN and University of Virginia in September 2007. Residents were informed of the study and invited to participate at introductory sessions held jointly by AKUHN program administrator and the pilot's community facilitator, author T.O.

### Interventions

#### Community of Learners (CoL)

The community facilitator (CF) helped residents to understand the basic elements of collaborative learning and behaviors expected of them as members of a CoL including self-regulation and facilitation of meetings; group commitment; information sharing; peer mentoring; and collective problem solving [2]. Residents met weekly for one hour to share and validate information gleaned during Internet teaching sessions, while collectively working through a series of clinical cases provided by the e-mentor. Two AKUHN Radiology faculty members and the community facilitator observed CoL and Internet sessions. In between meetings, residents shared learning and practice issues with each other and with the e-mentor by e-mail and a confidential Google Discussion Board.

#### Internet teaching sessions

Six two-hour sessions, facilitated by the e-mentor were held monthly using WebEx™Internet Conferencing facilities. Cases with radiologic images were presented and residents discussed their interpretation as a group; although typically at each conference, a resident also reviewed the consensus of findings of a case discussed at a CoL session.

### Evaluation methods

#### CoL event Log

Within 24 hours of CoL meetings, the community facilitator (CF) completed a CoL event log (see additional file 1) and posted observations derived from the event logs of resident activities, focusing on behaviors expected of members of a CoL, including regulation and facilitation of the meeting; interactivity and information sharing; peer mentoring; and collective problem solving.

#### Technology questionnaire

After each Internet session, residents, as part of a technology survey, (see additional file 2) provided their opinion on WebEx conferencing technology by completing a survey that determined its ease of use, reliability, and its support of learning during the session.

#### Resident exit survey

Residents responded to 10 statements (5 concerning their perceptions of CoL sessions and 5 related to WebEx conferences) using a Likert-like scale. Survey statements (see additional file 3) were shared with residents two months before the end of the study. They were invited to prepare stories in support of survey responses and recall them at the exit interviews.

#### Resident exit interviews

Each resident participated in a 20 minute semi-structured telephone interview during which they were invited to expand on their responses to exit survey statements, and relate stories they had prepared in support of them. Residents were given codes to ensure anonymity during telephone interviews.

#### Faculty exit interviews

Community Facilitator (T.O), e-Mentor (W.E.B.), AKUHN program administrator and 2 faculty members who attended CoL and WebEx conferences were invited to express their perceptions of the pilot in separate open-ended interviews. Resident and faculty telephone interviews were conducted and recorded by author F.M., located in Chicago, USA.

### Data Analysis

Quantitative analysis was limited to calculating mean values of scores derived from survey results. CF's observations derived from CoL log entries and data from interviews were transcribed verbatim and analyzed qualitatively, identifying and coding themes. Preliminary analyses were undertaken by I.J.P. and results reviewed by authors, AKUHN faculty and program administrator. The process was iterative as the group read, interpreted, discussed and revised interpretation of the data.

## Results

All 10 residents in AKUHN Radiology program volunteered for the e-mentoring pilot and signed consent forms: 7 residents (2 in 2^nd ^and 3^rd ^years, respectively, and 3 in 1^st ^year) participated from October 2007 to April, 2008. A further 3 residents who joined the 1^st ^year of the Radiology program in January 2008 participated in the pilot from January 2008 until its completion in April 2008. In all, residents attended 17 CoL and 6 WebEx conferences.

### Resident activities during CoL sessions

With exception of those on official leave, all residents attended 17 CoL sessions. Although present at all sessions CF completed event logs on only 10 due to demands on CF's time. There did not appear to be significant differences in resident behaviour at non-documented and documented sessions. Content analysis identified 39 entries supporting CoL behaviours: 6 describe how residents collectively decided CoL meeting format (self-regulation) - *".. residents ..... shared out the workload of seeking information (on cases). They agreed to share answers (at next meeting)" *(TO16); 7 entries describe how meeting facilitation rotated in a non-hierarchical group-initiated roster - "*Residents were led by a year-1 resident through the cases. .. Who would lead ... seemed to be arrived at prior to the session" *(TO15); 9 entries describe activities in support of peer mentoring - *"I began to see senior residents mentor junior ones in this meeting" *(TO4). Fifteen log entries describe how residents were observed sharing information about cases; 4 described how information obtained from the evidence-based literature mentioned during Internet sessions was used in conversations - *".. year 2 resident printed (and shared) the Depriest Score for ovarian masses.. pointed out by WEB (at an internet session)"*. (TO35). In addition, log entries described how discussion at early meetings focused entirely on clinical cases posted by the e-mentor, but later by residents involved personal learning issues in conversations. Log entries suggested that faculty members attending CoL meetings rarely participated in the conversations.

### Resident perception of WebEx conferencing technologies

Questionnaires were completed for 5 of 6 WebEx sessions: 6 residents completed questionnaires for sessions 1-3; and 8 for sessions 4 and 5. Technology problems experienced in the first session resulted in all 6 residents indicating that the WebEx technology was 'difficult to use' and learning was compromised by poor audio and visual transmission. The problems were corrected in session 2 onwards and this resulted in all or nearly all resident survey responses indicating that the WebEx system was 'easy to use' and audio and visual transmission was adequate and supported the learning experience.

### Resident perceptions of CoL and WebEx conferences as learning experiences

Likert-like scores assigned by residents to exit survey statements relating to perceived learning at CoL and WebEx sessions are summarized in Table 1. The scores indicate a high level of acceptance of CoL and Internet sessions as learning experiences. Residents were less in agreement that participating in CoL made them feel more comfortable discussing personal issues relating for instance to learning skills, service provision or home commitments. While 7 residents agreed with the statement, 2 responded "not sure" and 1 "disagreed". All 3 indicated at exit interviews that they were already discussing these issues with select colleagues before the CoL started. Residents provided additional information at the exit interviews including stories of personal experiences which validated their survey responses. The following are examples of statements made by residents at the interviews:

**Table 1:** Resident perceptions of learning in CoL and WebEx sessions

"Participation in CoL has enhanced my overall ability to learn in the residency program"	Mean Score 4.6
"Participation in CoL has given me confidence to ask questions and learn from my colleagues"	**4.2**

"Participating in CoL has helped me to apply new knowledge in my daily Radiology practice"	**4.6**

"Since participating in CoL I feel more comfortable asking a colleague for advice with a problem relating to my learning or service provision or home commitments"	**3.9**

"Participation in CoL has encouraged me to consult the medical evidence-based literature"	**4.7**

"WebEx sessions contributed positively to my overall residency experience"	**4.8**

"WebEx sessions helped me to learn a systematic approach to interpretation of diagnostic images"	**4.6**

"I was able to practice interpreting images and receive feedback in the WebEx sessions"	**4.6**

"I found the WebEx sessions helped me to learn"	**4.6**

"I used the teaching files (posted on FTP site) in between CoL and WebEx sessions"	**4.5**

Likert-like scores assigned by 10 residents to 10 statements posed in a survey and validated during exit interviews: 5 = strongly agree; 4 = agree; 3 = not sure; 2 = disagree; 1 = strongly disagree.

*"I now appreciate I can learn from colleagues. I was brought up to be taught by teachers" *[R1].

*"Initially I was hesitant in consulting my colleagues, but through the CoL I have learnt the need to share knowledge and different opinions *[R7]

*"There is a session where we were discussing .. antepartum hemorrhage in pregnancy, ... I am (now) able to advise clinicians better when they send requests to rule out placental abruption"*. [R4]

*"the .. session on radiation in pregnancy helped me to learn about..... current thinking of the cutoff levels. With this I was able to advise a doctor and patient who had been referred with RIF pain ....." *[R8]

*"during a CT round, a patient with an adrenal tumour had a dual phase scan. On remembering a particular session on adrenal masses, I was able to advise the technician ....." *[R9].

*CoL "helped ..(us) to be more cohesive and less competitive, and this was helpful" *[R4].

### Community facilitator perceptions of pilot

Two themes emerged from the exit interview with Community Facilitator (CF). First, residents were not familiar with the concept of CoL prior to the pilot, although 2 volunteered participating in informal group learning at university. There was a need to describe the potential benefits and behaviors expected of CoL members and encourage them to register for the pilot, but enthusiasm for CoL grew as they experienced the sessions. Second, during the interview, T.O. emphasized the high standard of teaching and commitment to resident learning demonstrated by the e-mentor and the observed positive impact on the residents. T.O. indicated his excitement in watching residents increase their level of interactivity and mentoring each other as the CoL sessions progressed. He stated, *"we are not just training experts here, we want to put good teachers in the community. Encouraging learning from each other, promotes a self-directed approach to learning"*.

### e-Mentor's perceptions of pilot

Three themes emerged from the exit interview with the e-mentor. First, W.E.B commended AKUHN residents for their knowledge and expertise and preparation of cases as well as their enthusiasm to learn and willingness to interact with colleagues during CoL and WebEx sessions. Second, he commented on the heavy time commitment involved in e-mentoring, indicating spending on average 3-5 hours per week selecting and posting cases and an additional 1-3 hours answering correspondence with residents. The third theme related to experiences with WebEx Internet technology. W.E.B. indicated that, in general, current Internet bandwidth to Nairobi was barely adequate for interactive teaching using diagnostic images. Simultaneous two-way video streaming, desirable for eye contact with residents, was not achieved due to limitations on bandwidth. W.E.B. praised AKUHN information technologists who played an essential supporting role during WebEx conferences.

### AKUHN faculty perceptions of pilot

Three themes emerged from analysis of separate interviews with AKUHN Program administrator and two faculty members who observed CoL and WebEx sessions. First, all three indicated that, in their opinion, the pilot had enriched the residency program by exposing residents to teaching and mentoring skills of a leading international teacher. Second, all 3 interviewees expressed concern that resident learning acquired during the pilot was not being applied to practice. The program administrator stated *"in retrospect, a major flaw in design may have been that involvement in the pilot was limited to only 2 of the 7 members of the teaching faculty. Other faculty members were not able to reinforce resident participation in the pilot as they were not involved"*.

Lastly, all 3 interviewees expressed interest in integration of e-mentoring into the AKUHN residency curriculum, suggesting two-weekly instead of weekly CoL sessions. They suggested that WebEx sessions should involve teachers at AKUHN and University of Virginia as learning facilitators. The interviewees recommended that a second multi-specialty study involving a larger number of residents and utilizing tools to objectively measure application of learning in practice should be considered.

### Study Limitations

Small numbers of residents in the pilot and its restriction to one discipline limit generalizability of results. Also, restricting the pilot to 6 months duration is a major limitation as it typically takes longer for communities to reach maturity [3]. Failure to keep teachers in the residency program aware of the CoL experiment was expressed as a "major flaw" by faculty members, as it was perceived to prevent teachers from assessing the impact of the study on resident performance outside CoL sessions. However, the results indicating resident acceptance of CoL as an effective method of learning suggests that a larger multidisciplinary study with objective assessment of learning is worthy of consideration.

## Discussion

Residents rapidly adopted CoL behaviors as described by Wenger et al, albeit with significant coaching by the pilot's community facilitator [3]. They collectively decided the format and facilitation of meetings; demonstrated willingness to learn from and mentor each other and collaboratively solve clinical problems. While these behaviors may be implicitly acquired by some residents in traditional residency programs who form natural CoLs with trusted colleagues, our experience supports Dornan [4] and Mennin [5] who suggest that formal education sessions be expanded to include CoL in which learning to practice is viewed as a social phenomenon and emerges through interactivity and self organization. The authors, based on reports of use of CoL in Higher education, [6] perceive learning in community as applicable to most if not all specialties, especially where multidisciplinary teams are solving complex patient problems.

Garrison's conceptual framework provides research evidence on which to build online and blended learning scenarios into residency education [2]. Although simultaneous group and individual learner evaluation presents its challenges, findings from research in higher education suggests that learning achievements in community are strengthened in programs that also place emphasis on individual accountability [9,10].

Residents in the pilot accepted CoL sessions as valid learning experiences. The e-mentor's decision to provide cases for residents to work through in CoL sessions proved an effective way of guiding new comers to group learning by focusing the discussion and promoting interactivity and collaborative problem solving. In our experience it takes time for a community to mature to the extent that members can freely discuss cases in their personal practice. The cases also provided opportunities for residents to practice image interpretation and problem solving skills taught during Internet sessions. The heavy time commitment in preparing teaching sessions indicated by the e-mentor is noted although updated course materials are reusable. The costs in time and other resources is likely to be offset by our finding of high value to learners, especially in residency programs where individualized teaching is limited due to heavy service commitments of clinical teachers and where experienced teachers are in short supply.

There were differences of opinion between program administrator and faculty members and residents concerning resident integration of learning into daily practice. While faculty felt knowledge translation from e-mentoring learning was limited, all residents in the exit interviews 'agreed or strongly agreed' with the statement: *"Participating in CoL has helped me to apply new knowledge in my daily Radiology practice" *and some provided stories in support of their responses. As is the case with community learning in general, repeated attendance at meetings is contributing evidence that residents benefited from interaction with peers.

Interactions between the e-mentor and residents were deemed by residents and faculty to be of an exceptionally high standard. Having an external teacher greatly contributed to the success of the pilot. It is generally agreed by administration and faculty that the post graduate program at AKUHN could in the future include CoL sessions as they compliment the program's initiatives to promote life-long, self-directed practice based learning among residents.

The pilot provided the planning team with a global vision for the future of radiology education. While there are still technological challenges to be overcome, the pilot highlighted the potential of e-mentoring as a means of expanding faculty and teaching materials in residency programs in developing countries.

## Conclusions

The pilot demonstrated resident acceptance of community building and collaborative learning as a valued learning experience, addressing one of the perceived barrier to its formal adoption in residency education curricula. It also highlighted the potential of e-mentoring as a means of expanding faculty and teaching materials in residency programs in developing countries.

## List of Abbreviations

AKUHN: Aga Khan University Hospital; Nairobi; CF: Community Facilitator; CoL: Community of Learners; CT: Computerized Tomography; F.M.: Fiona Miller; I.J.P: John Parboosingh; R: Resident; R.I.F.: Right Iliac Fossa; T.O.: Timona Obura; USA: United States of America; W.E.B.: William Brant

## Competing interests

The authors report no conflict of interest. The authors alone are responsible for the content and writing of the paper.

## Authors' contributions

Authors IJP, TO, FM and WEB developed, executed and evaluated the project. TO was Community Facilitator, WEB planned and executed Internet teaching sessions and produced the cases for community learning. FM coordinated the project and on its completion interviewed the residents and teachers. IJP and TO participated in the literature review. Preliminary analysis of results was undertaken by IJP while all authors participated in creating the final results. TO and IJP drafted the manuscript. All authors provided comments, read and approved the final manuscript.

## Authors' Information

TO is the Postgraduate Medical Education Director at the Aga Khan University in Nairobi with a special interest in collaborative learning. WEB is a Professor of Radiology based at the Department of Radiology, University of Virginia. FM is the Director of Research at the Radiological Society of North America. IJP is Professor Emeritus at the University of Calgary and Consultant of Community learning, Radiological Society of North America.

## Pre-publication history

The pre-publication history for this paper can be accessed here:

http://www.biomedcentral.com/1472-6920/11/3/prepub

## Supplementary Material

Additional File 1 **CoL session event log**. AKUHN E-Mentoring Project - Weekly CoL Sessions: Evaluation form. Observations on interactivity between residents and residents and faculty made by community facilitator at weekly CoL sessions and used to create a qualitative description of interactivity at sessions. Click here for file

Additional File 2 **E-mentoring learning technology survey form**. E-mentoring learning technology survey. Completed after each monthly WebEx teaching session, the survey provides resident perceptions of the impact of technology used in WebEx sessions, emails, Google discussion board and the FTP site where diagnostic images used at CoL sessions were archived. Click here for file

Additional File 3 **Resident Exit Survey and Interview Guide**. Evaluation of AKUHN E-Mentoring Pilot Project: Invitation to a survey and telephone interview. Resident perceptions obtained by responding to statements about learning experienced at CoL and WebEx sessions. Residents were provided with survey statements 2 months before end of pilot and submitted responses at time of exit telephone interviews. Residents were invited to collect stories in the 2 month period that validated their survey responses. Click here for file
