# Supplementary material for: Participating in a Community of Learners enhances resident perceptions of learning in an e-mentoring program: proof of concept
Source: BMC Med Educ. 2011 Jan 25;11:3. doi: 10.1186/1472-6920-11-3 (PMC3041783; doi:10.1186/1472-6920-11-3)
Supplement: Additional File 1 — CoL session event log. AKUHN E-Mentoring Project - Weekly CoL Sessions: Evaluation form. Observations on interactivity between residents and residents and faculty made by community facilitator at weekly CoL sessions and used to create a qualitative description of interactivity at sessions. [file 1472-6920-11-3-S1.DOC]

**AKUN E-Mentoring Project**

**Weekly CoL Sessions: Evaluation Form – PART 1**

**[**To be completed by Learner Facilitator at the completion of each session]

**DATE OF SESSION ……………………….**

| **Please CIRCLE one response for each of the following statements.** |
| --- |
| |  | **1. What was the overall direction of interactions during the session?** | | Most predominant interaction | |  | | Occurred  Occasionally | |  | | Least predominant interaction | | I cannot respond^1^ | | | --- | --- | --- | --- | --- | --- | --- | --- | --- | --- | --- | --- | --- | --- | --- | | a) Teacher to resident | | 5 | | 4 | | 3 | | 2 | | 1 | |  | |  | | b) Resident to teacher | | 5 | | 4 | | 3 | | 2 | | 1 | |  | |  | | c) Resident to resident | | 5 | | 4 | | 3 | | 2 | | 1 | |  | |  | |  | ^1^ Check this box if the statement is not relevant or you do not have sufficient information | | | | | | | | | | | | | | |

| **Please CIRCLE one response for each of the following statements.** |
| --- |
| | **2. What was the nature of the interaction between teacher and residents? The teacher:** | Used predominantly |  | Used  Occasionally |  | Not used  at all | I cannot respond^1^ | |  | | --- | --- | --- | --- | --- | --- | --- | --- | --- | | a) Provided information in response to questions from residents | 5 | 4 | 3 | 2 | 1 | |  |  | | b) Provided information before it was asked for | 5 | 4 | 3 | 2 | 1 | |  |  | | c) Waited for residents to offer their solutions before providing information | 5 | 4 | 3 | 2 | 1 | |  |  | | d) Provided supportive comments to encourage resident confidence in learning | 5 | 4 | 3 | 2 | 1 | |  |  | | e) Encouraged residents to answer each others’ questions | 5 | 4 | 3 | 2 | 1 | |  |  | | ^1^ Check this box if the statement is not relevant or you do not have sufficient information | | | | | | | | | |

| **Please CIRCLE one response for each of the following statements.** |
| --- |
| | **3. What was the nature of the interaction between residents? The residents:** | Used predominantly |  | Used  Occasionally |  | Not used  at all | I cannot respond^1^ | | --- | --- | --- | --- | --- | --- | --- | | a) Posed questions to each other about the cases | 5 | 4 | 3 | 2 | 1 |  | | b) Provided answers or suggestions in response to clinical problems posed by each other | 5 | 4 | 3 | 2 | 1 |  | | c) Showed evidence of mentoring each other (eg., provided non-threatening help) | 5 | 4 | 3 | 2 | 1 |  | | d) Talked to each other about a personal concern related to the residency program or work | 5 | 4 | 3 | 2 | 1 |  | | ^1^ Check this box if the statement is not relevant or you do not have sufficient information | | | | | | | |

**AKUN E-Mentoring Project**

**Weekly CoL Sessions: Evaluation Form – PART 2**

**[**To be completed by Learner Facilitator at the completion of each session]

**DATE OF SESSION ……………………….**

| **Please CIRCLE one response for each of the following statements.** |
| --- |
| | 4. **What was the nature of the interaction between residents and teacher? The residents:** | I strongly disagree | I Disagree | I am  not sure | I Agree | I Strongly agree | I cannot respond^1^ | | --- | --- | --- | --- | --- | --- | --- | | a) Expected the teacher to dictate the conversation during the meeting | 5 | 4 | 3 | 2 | 1 |  | | b) Solely answered questions posed by the teacher, as in an oral examination | 5 | 4 | 3 | 2 | 1 |  | | c) Asked the teacher for new information after interacting with each other | 5 | 4 | 3 | 2 | 1 |  | | d) Asked for evidence in support of a teacher’s statement | 5 | 4 | 3 | 2 | 1 |  | | ^1^ Check this box if the statement is not relevant or you do not have sufficient information | | | | | | | |

| **Please CIRCLE one response for each of the following statements.** |
| --- |
| | **5. Overall perceptions of the Learning Facilitator – in the opinion of the Learner Facilitator:** | I strongly Disagree | I disagree | I am  not sure | I agree | I strongly agree | I cannot respond^1^ | | --- | --- | --- | --- | --- | --- | --- | | a) The residents felt their learning needs were met | 5 | 4 | 3 | 2 | 1 |  | | b) The residents felt the learning environment was non-threatening | 5 | 4 | 3 | 2 | 1 |  | | c) The teachers enjoyed their experience | 5 | 4 | 3 | 2 | 1 |  | | d) Interaction between residents and teachers enhanced community building | 5 | 4 | 3 | 2 | 1 |  | | ^1^ Check this box if the statement is not relevant or you do not have sufficient information | | | | | | | |
