# Supplementary material for: Participating in a Community of Learners enhances resident perceptions of learning in an e-mentoring program: proof of concept
Source: BMC Med Educ. 2011 Jan 25;11:3. doi: 10.1186/1472-6920-11-3 (PMC3041783; doi:10.1186/1472-6920-11-3)
Supplement: Additional File 2 — E-mentoring learning technology survey form. E-mentoring learning technology survey. Completed after each monthly WebEx teaching session, the survey provides resident perceptions of the impact of technology used in WebEx sessions, emails, Google discussion board and the FTP site where diagnostic images used at CoL sessions were archived. [file 1472-6920-11-3-S2.DOC]

Please comment on your experience of the following technologies as part of the e-mentoring program.

| Tool |  | Agree | Disagree | Don’t know |
| --- | --- | --- | --- | --- |
| WebEx  (conferencing software for the monthly 2-hour sessions with Dr. Brant) | Was easy to use. |  |  |  |
|  | Worked reliably |  |  |  |
|  | Supported my learning |  |  |  |
| Discussion Board | Was easy to use |  |  |  |
|  | Worked reliably |  |  |  |
|  | Supported my learning |  |  |  |
| Email | Was easy to use |  |  |  |
|  | Worked reliably |  |  |  |
|  | Supported my learning |  |  |  |
| FTP | Was easy to use |  |  |  |
|  | Worked reliably |  |  |  |
|  | Supported my learning |  |  |  |

Please add comments you have regarding any of these tools:

Type comments here:
