# Supplementary material for: Participating in a Community of Learners enhances resident perceptions of learning in an e-mentoring program: proof of concept
Source: BMC Med Educ. 2011 Jan 25;11:3. doi: 10.1186/1472-6920-11-3 (PMC3041783; doi:10.1186/1472-6920-11-3)
Supplement: Additional File 3 — Resident Exit Survey and Interview Guide. Evaluation of AKUHN E-Mentoring Pilot Project: Invitation to a survey and telephone interview. Resident perceptions obtained by responding to statements about learning experienced at CoL and WebEx sessions. Residents were provided with survey statements 2 months before end of pilot and submitted responses at time of exit telephone interviews. Residents were invited to collect stories in the 2 month period that validated their survey responses. [file 1472-6920-11-3-S3.DOC]

**EVALUATION OF AKUHN E-MENTORING PILOT PROJECT:**

**Invitation to a survey and telephone interview**

Your interview is scheduled for [DATE & TIME]

Please dial in FIVE MINUTES before your interview

1-800……………………

Please state your Evaluation Identification Number [… ……………]

Dear [NAME],

Thank you for participating in the evaluation of AKUN E-Mentoring Pilot Project.

**In preparation for your interview**

You are invited to review the enclosed **TABLE OF STATEMENTS** about the Project as often as you can in the coming month as you will be requested to express your views on these statements during your interview.

While thinking about each statement, please make notes in your learning log or in the Table to remind yourself of any specific experiences that validate your response.

**Please be prepared to tell stories at the interview that describe personal experiences on these issues.**

On the day of the interview you are requested to use the Likert scale to record your responses to each statement and bring the Table of Statements and your learning log to the interview.

**The Interview**

**You are reminded to bring the Table of Statements and your Learning Log and any notes that will help you recall your stories to the interviewer.**

Your interview will take approximately 20 minutes. After dialing in, please state your ID number.

The interviewer will not see your learning log. You will be encouraged to use the learning log and your notes to jog your memory of a case or story that exemplifies or validates any comments you make during the interview.

Opinions expressed at the interview are greatly enhanced if you can describe a related case or an experience in the form of a short story.

**You will be asked to leave the Table of Statements with the completed Likert Scale in Dr Rees’ office after the interview.**

Yours sincerely

Timona Obura MD

Community Facilitator

AKUHN E-Mentoring Pilot

**TABLE OF STATEMENTS [PAGE 1 of 2]**

**Please insert your ID number here ………………………………..**

| A. Your opinions of the Community of Learners (AKUN CoL) | | | | | | |
| --- | --- | --- | --- | --- | --- | --- |
| | **Please CIRCLE one response** for each of the following statements. Please bring to the interview a story that confirms or validates your responses. | Strongly  Agree | Agree | Not  sure | Disagree | Strongly disagree | I have a story to tell | | --- | --- | --- | --- | --- | --- | --- | | 1. Participation in CoL has enhanced my overall ability to learn in the residency program  **Record your notes about a story:** | 5 | 4 | 3 | 2 | 1 | 🞎 | | 2. Participation in CoL has given me confidence to ask questions and learn from my colleagues  **Record your notes about a story:** | 5 | 4 | 3 | 2 | 1 | 🞎 | | 3. Participating in CoL has helped me to apply new knowledge in my daily Radiology practice  **Record your notes about a story:** | 5 | 4 | 3 | 2 | 1 | 🞎 | | 4. Since participating in CoL I feel more comfortable asking a colleague for advice with a problem relating to my learning or service provision or home commitments.  **Record your notes about a story:** | 5 | 4 | 3 | 2 | 1 | 🞎 | | | | | | | |
| 5. Participation in the CoL has encouraged me to consult the medical literature to answer the questions posed or to or clarify the teaching cases reviewed.  **Record your notes about a story:** | 5 | 4 | 3 | 2 | 1 | 🞎 |

**TABLE OF STATEMENTS [PAGE 2 of 2]**

**Please insert your ID number here ………………………………..**

| B. Your opinions of the monthly WebEx teaching sessions |
| --- |
| | **Please CIRCLE one response** for each of the following statements. Where possible please bring to the interview a story that confirms and validates your response. | Strongly  Agree | Agree | Not  sure | Disagree | Strongly disagree | I have a story to tell | | --- | --- | --- | --- | --- | --- | --- | | 1. WebEx sessions contributed positively to my over all residency experience  **Record your notes about a story:** | 5 | 4 | 3 | 2 | 1 | 🞎 | | 2. WebEx sessions helped me to learn a systematic approach to the interpretation of diagnostic images  **Record your notes about a story:** | 5 | 4 | 3 | 2 | 1 | 🞎 | | 3. I was able to practice interpreting images and receive feedback in the WebEx sessions  **Record your notes about a story:** | 5 | 4 | 3 | 2 | 1 | 🞎 | | 4. I found the sessions helped me to learn  **Record your notes about a story:** | 5 | 4 | 3 | 2 | 1 | 🞎 | | 5. I used the teaching files in between sessions  **Record your notes about a story:** | 5 | 4 | 3 | 2 | 1 | 🞎 | |
